# Evaluation of Chemical Changes in Laboratory-Induced Colistin-Resistant *Klebsiella pneumoniae*

**DOI:** 10.3390/ijms22137104

**Published:** 2021-07-01

**Authors:** Agata Pruss, Paweł Kwiatkowski, Łukasz Łopusiewicz, Helena Masiuk, Peter Sobolewski, Karol Fijałkowski, Monika Sienkiewicz, Adam Smolak, Stefania Giedrys-Kalemba, Barbara Dołęgowska

**Affiliations:** 1 Department of Laboratory Medicine, Chair of Microbiology, Immunology and Laboratory Medicine, Pomeranian Medical University in Szczecin, Powstańców Wielkopolskich 72, 70-111 Szczecin, Poland; agata.pruss@pum.edu.pl (A.P.); barbara.dolegowska@pum.edu.pl (B.D.); 2 Department of Diagnostic Immunology, Chair of Microbiology, Immunology and Laboratory Medicine, Pomeranian Medical University in Szczecin, Powstańców Wielkopolskich 72, 70-111 Szczecin, Poland; 3 Center of Bioimmobilisation and Innovative Packaging Materials, Faculty of Food Sciences and Fisheries, West Pomeranian University of Technology Szczecin, Janickiego 35, 71-270 Szczecin, Poland; lukasz.lopusiewicz@zut.edu.pl; 4 Department of Medical Microbiology, Chair of Microbiology, Immunology and Laboratory Medicine, Pomeranian Medical University in Szczecin, Powstańców Wielkopolskich 72, 70-111 Szczecin, Poland; h.masiuk@op.pl (H.M.); kalemba@mp.pl (S.G.-K.); 5 Department of Polymer and Biomaterials Science, Faculty of Chemical Technology and Engineering, West Pomeranian University of Technology Szczecin, Piastów 45, 70-311 Szczecin, Poland; psobolewski@zut.edu.pl; 6 Department of Microbiology and Biotechnology, West Pomeranian University of Technology Szczecin, Piastów 45, 70-311 Szczecin, Poland; karol.fijalkowski@zut.edu.pl; 7 Department of Allergology and Respiratory Rehabilitation, Medical University of Łódź, Żeligowskiego 7/9, 90-752 Łódź, Poland; monika.sienkiewicz@umed.lodz.pl; 8 Microbiological Laboratory, Independent Public Clinical Hospital No. 1 in Szczecin, Unii Lubelskiej 1, 71-252 Szczecin, Poland; admsmolak@gmail.com

**Keywords:** colistin resistance, FTIR spectroscopy, Raman spectroscopy, ζ-potential, *Klebsiella pneumoniae*

## Abstract

This study evaluates the electrical potential and chemical alterations in laboratory-induced colistin-resistant *Klebsiella pneumoniae*, as compared to the susceptible strain using spectroscopic analyses. The minimal inhibitory concentration (MIC) of colistin, ζ-potential and chemical composition analysis of *K. pneumoniae* strains are determined. The results obtained for the *K. pneumoniae*^Col-R^ with induced high-level colistin resistance (MIC = 16.0 ± 0.0 mg/L) are compared with the *K. pneumoniae*^Col-S^ strain susceptible to colistin (MIC = 0.25 ± 0.0 mg/L). Fourier transform infrared (FTIR) and Raman spectroscopic studies revealed differences in bacterial cell wall structures and lipopolysaccharide (LPS) of *K. pneumoniae*^Col-R^ and *K. pneumoniae*^Col-S^ strains. In the beginning, we assumed that the obtained results could relate to a negative charge of the bacterial surface and different electrostatic interactions with cationic antibiotic molecules, reducing the affinity of colistin and leading to its lower penetration into *K. pneumoniae*^Col-R^ cell. However, no significant differences in the ζ-potential between the *K. pneumoniae*^Col-R^ and *K. pneumoniae*^Col-S^ strains are noticed. In conclusion, this mechanism is most probably associated with recognisable changes in the chemical composition of the *K. pneumoniae*^Col-R^ cell wall (especially in LPS) when compared to the susceptible strain.

## 1. Introduction

*Klebsiella pneumoniae* is a widespread Gram-negative bacterium (a member of the *Enterobacteriaceae* family) that can colonise mucous membranes, as well as other niches of humans and animals [1]. Major virulence factors produced by this bacterium include a polysaccharide capsule providing protection against phagocytosis and opsonisation, as well as lipopolysaccharide (LPS), whose composition increases the immune system response and makes *Klebsiella* resistant to complement-mediated killing [2]. Due to its ability to survive in inappropriate conditions, *K. pneumoniae* is one of the most common causes of healthcare-associated infections. Moreover, *K. pneumoniae* strains acquire resistance to a large number of antibiotics through multifaceted mechanisms. The occurrence of extended-spectrum β-lactamases (ESBL) represents the most common resistant phenotype, whereas carbapenemase-producing species are an important source of concern. The incidence of nosocomial infections caused by multi-drug resistant *K. pneumoniae* has increased in recent years and is associated with a high level of mortality [3]. Therefore, clinicians have returned to the older generations of antibiotics.

Colistin (polymyxin E) represents peptide antibiotics classified as a member of the polycationic group [4]. It was discovered in 1949 and described as nonribosomal peptides synthesised by *Bacillus polymyxa* subsp. *colisitnus* [5,6]. Colistin, an antibacterial agent representing an older group of antimicrobials, remained the last-resort treatment option of infections caused by carbapenemase-producing *Enterobacteriaceae* [7].

According to available data, colistin resistance demonstrated by *K. pneumoniae* is increasingly reported worldwide [8]. Colistin resistance is strongly related to altered LPS consisting of lipid A (4’-phosphoethanolamine) in the bacterial outer membrane. This mechanism is mostly associated with the mutations in chromosomal DNA related to LPS biosynthesis and plasmid-transferred mobilised colistin resistance gene (*mcr-1*) present in certain species of *Enterobacteriaeceae* (most commonly *Escherichia coli*) [9]. Nevertheless, the genetic background of colistin resistance in *K. pneumoniae* has not been fully recognised. 

Fourier transform infrared (FTIR) and Raman spectroscopies have been shown to be a useful method for investigating biological macromolecules, as well as the complex biological systems, such as tissues and cells [10,11,12,13,14,15,16,17,18]. Detection and identification of microorganisms using spectroscopic techniques are promising methods, due to their sensitivity, simplicity, and time- and cost-effectiveness. Spectroscopic tools provide not only rapid identification, but also investigates the microorganisms in their intact state. They also appear to be a very promising tool to study microbial metabolism, antibiotic susceptibility, as well as other interactions with drugs [10]. There is a great potential for spectroscopic methods in tandem with appropriate mathematical tools for simple and rapid discrimination and identification of various microorganisms [19]. Moreover, the chemical composition of the samples can also be visualised simultaneously. Hence, FTIR and Raman spectroscopies provide an important aid in the understanding of complex chemical processes during bacterial development, including antibiotic resistance development. According to available data, the analysis of colistin-resistant *K. pneumoniae* using FTIR and Raman spectroscopies has not been performed so far.

The present study evaluates the electric potential and chemical alterations in the colistin-resistant laboratory-induced *K. pneumoniae* as compared to the susceptible strain using the ζ-potential, as well as FTIR and Raman spectroscopies.

## 2. Results and Discussion

Over time, colistin (a potentially a nephrotoxic antibiotic) has been replaced by less toxic antimicrobial agents, but it still remains as a reserved treatment option [20]. Reintroduction of colistin into treatment has been associated with the increasing prevalence of life-threatening infections caused by Gram-negative rods exhibiting simultaneous resistance to quinolones, aminoglycosides and all β-lactams [21]. It is worth mentioning that colistin resistance is increasingly observed among multiple representatives of *Enterobacterales* and non-fermentative rods, which is attributed to drug overuse. Colistin-resistant K. pneumoniae is becoming a global health concern and infects patients from all around the world. According to available data, the highest rate of colistin resistance was reported among 10–20% of clinical strains in Greece in 2010–2011 [22,23]. Slightly lower percentages of colistin-resistant *K. pneumoniae* were reported in South Korea (6.8%), Singapore (6.3%), or Canada (2.9%) [24,25,26]. It is also not surprising that about 22–50% of those strains were isolated from infected patients’ hospitalised in intensive care units [27].

**Figure 1:**
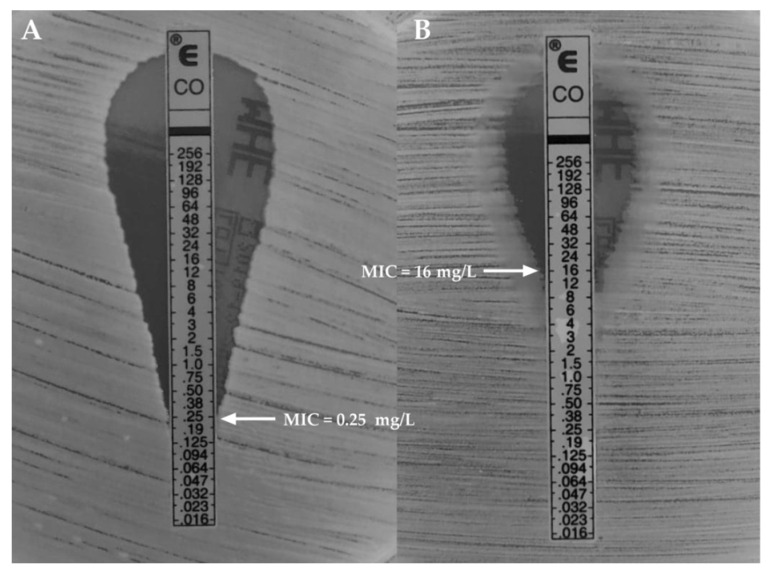
Minimal inhibitory concentration (MIC) of colistin-susceptible ((**A**)—*K. pneumoniae^Col-S^*) and induced high-level colistin-resistance ((**B**)—*K. pneumoniae^Col-R^*) *K. pneumoniae* strains.

In the current study, we proved that after 48 hours of exposure to various colistin concentrations, the minimal inhibitory concentration (MIC) value for *K. pneumoniae* ATCC^®^ BAA 1705™ was 0.25 ± 0.0 mg/L and remained in the susceptible range. After the next 48 hour-exposure to obtained MICs of colistin, the MIC value increased and was in the range for the high-level resistance (MIC = 16.0 ± 0.0 mg/L, designated as *K. pneumoniae^Col-R^*). Simultaneously, the MIC for control strain (non-exposed to colistin) remained unchanged (MIC = 0.25 ± 0.0 mg/L, designated as *K. pneumoniae^Col-S^*) (Figure 1). Moreover, the resistance in the *K. pneumoniae^Col-R^* strain persisted even after reseeding.

**Figure 2:**
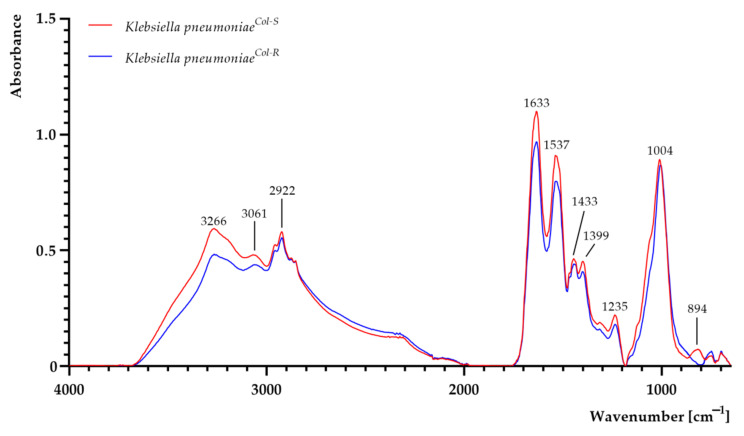
FTIR spectra of colistin-susceptible (*K. pneumoniae^Col-S^*) and laboratory-induced high-level colistin-resistant *K. pneumoniae* (*K. pneumoniae^Col-R^*) strains.

The FTIR spectra of *K. pneumoniae^Col-S^* and *K. pneumoniae^Col-^*^R^ cells are shown in Figure 2. Areas of all peaks are listed in Supplementary Table S1. The differences were particularly observed in the complex spectral region at 1700–1200 cm^−1^, assigned to amide, mixed and phospholipid regions, where many types of vibrations are present (including CH_2_ bending, COO^−^ stretching and PO_2−_ stretching) [28,29,30]. The differences were also present in the 1200–650 cm^−1^ region, which corresponds to stretching and bending vibrations of C–O–C and C–OH groups, as well as the C–O ring of the polysaccharide region attributed to carbohydrates (polysaccharides) in the bacterial cell wall [28,29,30,31,32]. At 3266 cm^−1^, the absorbance value of *K. pneumoniae^Col-S^* was higher than that of *K. pneumoniae^Col-R^*, suggesting more amino and hydroxyl groups in the *K. pneumoniae^Col-S^* strain [33]. In *K. pneumoniae^Col-R^*, the growth of absorbance at band 2922 cm^−1^ (attributed to asymmetric and symmetric stretching, vibrations of CH_2_ and CH_3_ groups) was observed [33,34]. At 1633 cm^−1^, the absorbance value was higher in the sample of *K. pneumoniae^Col-S^*, attributed to C=O and C–N of proteins [28,29,33]. In the amide II region, whose bands are attributed to the asymmetric bending of methyl and carboxyl groups in proteins and lipids, as well as stretching C–N vibrations of cytosine-guanine pairs, the absorbance slightly increased at 1443 cm^−1^ and at 1399 cm^−1^ in the *K. pneumoniae^Col-S^* sample [28,29,33]. An increase in absorbance at 1235 cm^−1^ was imputed to asymmetric stretching of the phosphodiester backbone and deformation vibration of C=O carboxylic acids [13,14,33]. At 1004 cm^−1^ attributed to stretching, OH coupled with bending CO of capsule and peptidoglycan polysaccharides in *K. pneumoniae^Col-S^* was observed. At wavenumbers attributed to stretching CO of polysaccharides, a decline of absorbance was observed in *K. pneumoniae^Col-R^* [15,33]. The noticeably increased absorbance in the narrow region centred at 894 cm^−1^, assignable to C–O–C and C–O–P symmetric stretching in cell wall oligosaccharides and polysaccharides in *K. pneumoniae^Col-S^*, was also reported [14]. The increased absorbance in the fingerprint region 820−650 cm^−1^ (attributed to N-containing bioligands) in *K. pneumoniae^Col-R^* was observed [13,29,33,35].

**Figure 3:**
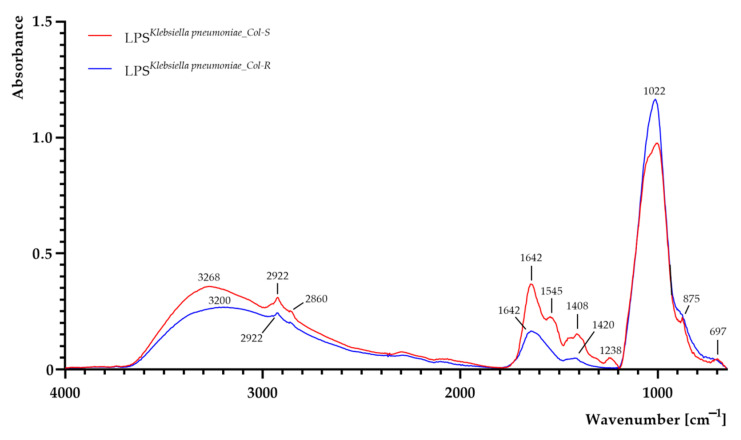
FTIR spectra of lipopolysaccharide (LPS) extracted from colistin-susceptible (LPS*^K. pneumoniae^*^_*Col-S*^) and laboratory-induced high-level colistin-resistant (LPS*^K. pneumoniae^*^_*Col-R*^) *Klebsiella pneumoniae* strains.

The FTIR spectra of LPS samples extracted from both *K. pneumoniae^Col-S^* and *K. pneumoniae^Col-R^* are shown in Figure 3. Areas of all peaks are listed in Supplementary Table S1. LPS consists of O-polysaccharide, core polysaccharide and lipid A [36]. As can be seen, a shift and a decrease in band intensity from 3268 cm^−1^ to 3200 cm^−1^ (O–H and N–H groups) were observed in LPS*^K. pneumoniae_^^Col-R^* sample [32]. Moreover, a slight decrease in absorbance was observed at 2922 cm^−1^ (methylene groups) and 2860 cm^−1^ (assigned to the –CH stretching region (–CH_3(asymm)_, –CH_3(symm)_ and –CH modes), suggesting fatty acid chain alterations in lipid A moieties, as CH_2_ (methylene) groups are sensitive to structural changes in fatty acid chains [32,37]. The absorption peaks between 1800 cm^−1^ and 1000 cm^−1^ are assigned to O-antigen [32]. A decrease in absorbance for LPS*^K. pneumoniae_^^Col-R^* sample was noticed and can be linked with less O-antigen in the colistin-resistant strain [32]. The formation of peaks at 1642 cm^−1^, 1408 cm^−1^ and 1255 cm^−1^ is typically assigned to uronic aids associated with O-acetyl groups [32], and decrease in absorption at those wavenumbers was observed in LPS*^K. pneumoniae_^^Col-R^* sample. The intense band in the region of 1100–900 cm^−1^ can be attributed to the carbohydrate C–O–C ring absorption, as well as *_ν_*C–C and *_ν_*C–O of the glycosidic linkage [32], and the increase in intensity was noticed for LPS*^K. pneumoniae_^^Col-R^* sample. In addition, a signal at 1238 cm^−1^ (assigned to the _ν_C–O of carboxylic acids) was noticed in LPS*^K. pneumoniae_^^Col-S^* sample, suggesting that the LPS polymer of the colistin-sensitive strain was acidic [32], contrary to LPS of the colistin-resistant strain, which lacked a peak at this wavenumber.

**Figure 4:**
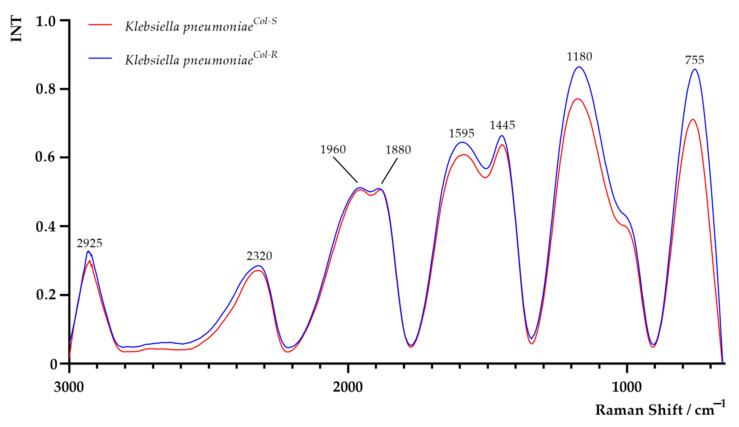
Raman spectra of colistin-susceptible (*K. pneumoniae^Col-S^*) and laboratory-induced high-level colistin-resistant *Klebsiella pneumoniae* (*K. pneumoniae^Col-R^*) strains.

The Raman spectra of *K. pneumoniae^Col-S^* and *K. pneumoniae^Col-R^* cells are shown in Figure 4. Areas of all peaks are listed in Supplementary Table S1. Regarding the Raman analysis, a noticeably higher band between 650 and 900 cm^−1^ (centred around 755 cm^−1^) in the *K. pneumoniae^Col-R^* sample was observed. This band is related to DNA and RNA and attributed to adenine containing species, to a mode of adenine ring breathing in DNA and DNA phosphodiester stretching, out-of-plane ring breathing modes of tyrosine, and to the C–O–C stretching vibration of 1,4 glycosidic link in carbohydrates [11,12]. The region between 1800 cm^−1^ and 900 cm^−1^ was characterised by a series of emission bands from amides, lipids and carbohydrates [11]. The band at 1180 cm^−1^ in *K. pneumoniae^Col-R^* was higher than in *K. pneumoniae^Col-S^*, related to C–O–C and =C–C= antisymmetric stretching in aliphatic esters and glycosidic link of carbohydrates [11]. At 1445 cm^−1^_,_ the higher signal intensity in *K. pneumoniae^Col-R^*—most probably attributed to CH_2_ vibrations of membrane lipids and polysaccharides, such as poly-N-acetylglucosamine, was reported [11,16]. In the region between 2800 and 3000 cm^−1^, attributed to symmetric and antisymmetric stretching of CH_2_ and CH_3_ in lipids, fatty acids, proteins and carbohydrates, a slightly more intense signal in *K. pneumoniae^Col-R^* was observed [11,34].

**Figure 5:**
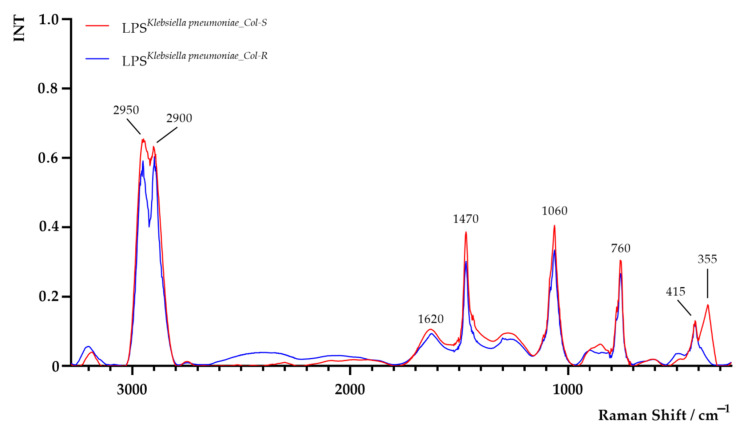
Raman spectra of lipopolysaccharide (LPS) extracted from colistin-susceptible (LPS*^K. pneumoniae^*^_*Col-S*^) and laboratory-induced high-level colistin-resistant (LPS*^K. pneumoniae^*^_*Col-R*^) *Klebsiella pneumoniae* strains.

The Raman spectra of LPS extracted from both *K. pneumoniae^Col-S^* and *K. pneumoniae^Col-R^* are shown in Figure 5. Areas of all peaks are listed in Supplementary Table S1. In the Raman spectrum of LPS*^K. pneumoniae_Col-S^* sample, significant differences in peaks intensity at 1470 cm^−1^, 1060 cm^−1^ and 760 cm^−1^ and an additional peak at 355 cm^−1^ were observed. The obtained results suggest probable alterations in the LPS chemical composition, with special emphasis on CH_2_-deformation vibration, C–O–H and C–O–C vibrations and O–P–O stretching (from lipid and polysaccharide parts of LPS which may alter interactions of LPS hydrophobic groups with the surrounding) [38,39,40].

Activation of multiple LPS-modifying genes is involved in polymyxin resistance in Gram-negative bacteria. At the genetic level, colistin resistance in *K. pneumoniae* is associated with the presence of the *mcr-1* gene. Both mutation or inactivation of the other gene, *mgrB,* has been found to play a prominent role in colistin resistance in *K. pneumoniae* [41]. Moreover, silent mutations, point mutations, insertions and deletions in the genes related to LPS synthesis have been reported in colistin-resistant *K. pneumoniae* isolated from blood infections, which may contribute to accelerating the antimicrobial resistance [42]. As stated, the exact downstream between genes and intracellular factors, which conferred colistin resistance, remains not fully recognised.

The colistin molecule consists of a cationic cyclic decapeptide linked to a fatty acid (6-methyl-octanoic or 6-methyl-eptanoic acids) chain through the α-amide linkage [43]. The target of antimicrobial activity of colistin is the bacterial cell membrane. The initial association of colistin with the bacterial membrane occurs through electrostatic interactions between cationic polypeptide (colistin) and anionic LPS molecules in the outer membrane of the Gram-negative bacteria, leading to disturbances to the cell wall stability. Colistin displaces magnesium and calcium ions (which are playing a pivotal role in the LPS molecule stabilisation) from the negatively charged LPS, leading to local disturbances in the outer membrane. As a result, increased permeability of the cell envelope and formation of pores lead to leakage of cell contents, and subsequently, cell death occurs [43,44,45]. Other authors observed that the resistance of other Gram-negative bacteria (such as *Pseudomonas* spp.) is associated with bacterial outer membrane alterations, i.e., reduction in LPS and specific outer membrane protein levels, reduction in cell envelope Mg^2+^ and Ca^2+^ contents and lipid alterations [46,47]. The protective function of the outer membrane mainly relies on the presence of polyanionic LPS, which limits the penetration of hydrophobic and/or large antibiotics. The initial binding of colistin to the bacterial surface depends on the electrostatic interaction between the positively charged polycationic ring and the negatively charged phosphate group of lipid A of LPS.

In the current study, we also proved that for both strains (*K. pneumoniae^Col-S^* and *K. pneumoniae^Col-R^*), hydrodynamic size measurements yielded similar, monomodal size distributions with PDI < 0.3 and similar calculated hydrodynamic diameters (~2.3 µm) (Table 1). For rod-shaped *K. pneumoniae,* these values represent diameters of hard spheres with the measured diffusion coefficient, and our results are in good agreement with the prior particle analysis and microscopy data for *K. pneumoniae* [48]. As regards ζ-potential, the two strains were also similar, with both exhibiting average ζ-potential values of approx. –13 mV (in saline). However, these data were not statistically significant (*p* > 0.05). These values are in good agreement with a prior study focused on colistin-susceptible and colistin-resistant *K. pneumoniae* isolates, which also noted no significant differences in the ζ-potential between the two isolates [49]. 

Resistance to colistin has several molecular mechanisms that have been characterised in various bacterial species [45]. The resistance may result from: (i) Specific modification of outer membrane porins and reductions in the overall negative charge of the LPS, (ii) overexpression of efflux pump systems, and (iii) overproduction of capsular polysaccharide [43,45]. More advanced studies were conducted to clarify potential mechanisms of colistin resistance in Acinetobacter baumannii by combining laboratory-induced resistance with a high-throughput genome. In this context, researchers revealed that colistin resistance in A. baumannii can be generated rapidly in laboratory conditions [50].

It is worth emphasising that our findings represent a significant clinical implication. To date, multi-drugs resistance in nosocomial strains has been noticed as an environmental issue, and single cases were considered as successfully treated, if managed consistently with relevant recommendations, e.g., carbapenems for ESBL-positive Gram-negative rods. Furthermore, resistance development challenged drug use policy and the management of other patients. Our study indicated that colistin resistance is inducible in vitro in a short timeframe, so that analogously it may appear in a patient during a single therapy course (which targets primarily susceptible bacterial strain), consequently, resulting in therapeutic failure.

## 3. Materials and Methods 

### 3.1. Bacterial Strain and Growth Conditions

The *K. pneumoniae* ATCC^®^BAA1705™ was used in this study. Each time, prior to the experiment, the strain was cultivated for 18 h at 37 °C in an aerobic atmosphere on Columbia agar with 5% sheep blood (bioMérieux, Warsaw, Poland).

### 3.2. Determination of Minimal Inhibitory Concentration (MIC) of Colistin Against K. pneumoniae ATCC^®^BAA1705™

The MIC of colistin was determined by the broth microdilution test using ComASP Colistin 0.25−16 mg/L MIC assay (Diagnostics, New Ash Green, England) according to the current European Committee on Antimicrobial Susceptibility Testing interpretative criteria [51], and manufacturer’s protocol. All tests were performed in triplicate.

### 3.3. Colistin Resistance Induction

The colistin resistance in *K. pneumoniae* ATCC^®^ BAA 1705™ was induced using the ComASP Colistin 0.25−16 mg/L MIC test. One colony of *K. pneumoniae* ATCC^®^ BAA 1705™ was transferred from pure 18-24 h culture to sterile saline and adjusted to match the turbidity standard of 0.5 McFarland units. Then, 60 µL of freshly prepared bacterial suspension was inoculated into suspension media (attached to the test) and mixed. The bacterial suspension (100 µL) was added to each well of a microplate and incubated for 48 h at 37 °C in aerobic conditions. After incubation, the MIC value was determined in the first well without the appearance of growth (measured by the naked eye) in comparison to the growth-control well. This stage was designated as “passage 0”. 

In the next stage, the strain was transferred from the first MIC well (“passage 0”) and subjected to another 48 h colistin exposure cycle under the same conditions. This stage was designated as “passage I”. *K. pneumoniae* ATCC^®^BAA1705™ with acquired colistin resistance was designated as *K. pneumoniae^Col-R^*. Simultaneously, the control was conducted under the same conditions without colistin, and *K. pneumoniae* ATCC^®^BAA1705™ was designated as *K. pneumoniae^Col-S^*. Furthermore, the MIC values for both strains were confirmed using the E-test method in triplicate.

### 3.4. LPS Extraction

LPS was extracted from both *K. pneumoniae^Col-S^* and *K. pneumoniae^Col-R^* strains using the hot phenol-water method according to the previously described protocol [52] with some modifications. Bacterial suspensions (4 McFarland units) were centrifuged at 10,000× *g* for 5 min. The pellets were washed twice in PBS (0.15 M) containing 0.15 mM CaCl_2_ and 0.5 mM MgCl_2_. Then pellets were resuspended in 10 mL PBS and sonicated using a sonificator (UPS400S Ultrasonic Processor, Hielsher Ultrasound Technology, Teltow, Germany) for 10 min (0.7 cycle/min; 100% amplitude) on ice. The next step was the treatment with enzymes (Thermo Fisher Scientific, Waltham, MA, USA): proteinase K, DNase, and RNase to eliminate contaminating nucleic acids and protein. For extraction, proteinase K (100 µg/mL) was added to the cell mixture, and the tubes were incubated at 65 °C for 60 min. At the next step, the mixture was treated with RNase (40 µg/mL) and DNase (20 µg/mL) in the presence of 1 µL/mL chloroform (Sigma-Aldrich, Darmstadt, Germany) and incubated at 37 °C overnight. 

In the next stage, an equal volume of hot (70 °C) 90% phenol (Sigma-Aldrich, Darmstadt, Germany) was added to the mixtures followed by vigorous shaking at 65–70 °C for 15 min. Suspensions were then cooled on ice and transferred to 1.5 mL Eppendorf tubes, and centrifuged at 8500× *g* for 15 min. Supernatants were transferred to 15 mL conical centrifuge tubes, and phenol phases were re-extracted by 300 µL distilled water. Sodium acetate at 0.5 M final concentration and 10 volumes of 95% ethanol (Sigma-Aldrich, Darmstadt, Germany) were added to the extracts and samples were stored at –20 °C overnight to precipitate LPS. 

Next, tubes were centrifuged at 2000× *g* in 4 °C for 10 min, and the pellets were resuspended in 1 mL distilled water. Extensive dialysis against double distilled water at 4 °C was carried out until the residual phenol in the aqueous phases was eliminated. The final purified LPS product was lyophilised for 24 h (chamber pressure 0.190 mbar, shelf temp. T_min_ = –35 °C, T_max_ = 20 °C; condenser temp. –85 °C) in a Beta 2–8 LSCplus lyophiliser (Martin Christ Gefriertrocknungsanlagen GmbH, Osterode am Harz, Germany). The obtained samples were stored at 4 °C for further analyses.

### 3.5. Determination of Functional Groups in K. pneumoniae Cells and Extracted LPS Using FTIR and Raman Spectroscopies

In order to confirm the presence of particular chemical moieties in *K. pneumoniae^Col-S^* and *K. pneumoniae^Col-R^*, as well as in their LPS, FTIR and Raman spectroscopic analyses, were performed [18]. After 18 h cultivation of each strain at 37 °C on Columbia agar with 5% sheep blood, bacterial colonies were harvested, transferred to an Eppendorf tube, and washed three times using saline. Then, the samples were centrifuged at 5000× *g* for 5 min and dried for 24 h at 37 °C. The FTIR spectra of dried bacterial cell samples and freeze-dried LPS were obtained at room temperature by an attenuated total reflection FTIR spectrometer (Perkin Elmer Spectrophotometer 100, Waltham, MA, USA). The samples (100 mg) were then scanned at a range between 650 cm^−1^ and 4000 cm^−1^ (64 scans and 1 cm^−1^ resolution). The obtained spectra were normalised, baseline corrected, and analysed using SPECTRUM software (v10, Perkin Elmer Spectrophotometer, Waltham, MA, USA).

To obtain Raman spectra, the samples were analysed using the Raman spectrometer (RamanStation 400F, Perkin Elmer, Waltham, MA, USA) with point and shot capability and excitation laser source at 785 nm (to avoid fluorescence excitation), 100-micron spot size, and 4 scans (8 s exposition time). The obtained spectra were normalised, baseline corrected and analysed using SPECTRUM software (v10, Perkin Elmer, Waltham, MA, USA).

### 3.6. Dynamic Light Scattering Assay

Dynamic light scattering (DLS) was used to assess hydrodynamic size and ζ-potential of *K. pneumoniae^Col-S^* and *K. pneumoniae^Col-R^* strains. Measurements were performed using a Nanosizer ZS instrument (Malvern Panalytical Ltd., Malvern, UK) equipped with a 633 nm He-Ne laser. Bacterial cell suspensions (0.5 McFarland units) were prepared in saline (0.9%). Samples were prepared at room temperature, the instrument was pre-equilibrated at 25 °C, and all measurements were performed at 25 °C. The mean counting rate of all measurements was <500 kcps. For hydrodynamic size measurements, approx. 1 mL of suspension was pipetted into a disposable polystyrene cuvette following the manufacturer’s instructions. For each sample, 3–6 measurements were performed, each consisting of 15 runs, with automatic attenuation and position selection, and using a 173° scattering angle. The reported hydrodynamic size and polydispersity index (PDI) were derived from the Cumulant analysis using Zetasizer software (Malvern, UK) as the intensity-weighted mean particle diameter (z-Average Diameter) and broadness of the size distribution, respectively. For ζ-potential measurements, approx. 0.8 mL of the cell suspension was pipetted into a disposable folded capillary cell following the manufacturer’s instructions. For each sample, 3–6 measurements were performed, each consisting of 15 runs, with automatic attenuation and voltage selection. The data were analysed using Zetasizer software (Malvern, UK) in a monomodal mode and the Smoluchowski model (F(k⋅α) = 1.5) to obtain average ζ-potential values.

### 3.7. Statistical Analysis

All data were expressed as mean ± standard deviation (SD). The statistical analysis between the results obtained in the electrical potential was performed using StatSoft Statistica 13.0 (StatSoft Inc, Tulsa, OK, USA) and GraphPad Prism 8.0.1 (GraphPad Software, San Diego, CA, USA). To assess the differences between the examined parameters, the one-way analysis of variance (ANOVA) with the Tukey’s post hoc test was evaluated. Differences were considered significant at *p* < 0.05.

## 4. Conclusions

Based on the FTIR and Raman spectral analyses of *K. pneumoniae^Col-S^* and *K. pneumoniae^Col-R^*, it can be concluded that resistance to colistin may affect the chemical composition of bacterial cells. Those alterations particularly related to polysaccharides and peptidoglycan of the capsule, cell wall oligosaccharides and polysaccharides, as well as lipids of the bacterial membrane. In the beginning, we assumed that the obtained results could relate to a negative charge of the bacterial surface and different electrostatic interactions with cationic antibiotic molecules, reducing the affinity of colistin and leading to its lower penetration into *K. pneumoniae^Col-R^* cell. However, the ζ-potential proved no significant differences in the ion charge between the analysed isolates. The dynamics of resistance mechanism development is potentially an extreme challenge for therapeutic processes in combating infections caused by multi-drug resistant *K. pneumoniae* strains, as well as other Gram-negative bacteria. However, to determine the genetic background of the described resistance mechanism, further research is required.

## Figures and Tables

**Table 1:** Dynamic light scattering results of colistin-susceptible (*K. pneumoniae^Col-S^*) and laboratory-induced high-level colistin-resistant (*K. pneumoniae^Col-R^*) *Klebsiella pneumoniae* strains.

*K. pneumoniae* Strains	Hydrodynamic Diameter [nm]	Polydispersity Index (PDI)	ζ-Potential [mV]
*K. pneumoniae^Col-S^*	2340 ± 40 ^a^	0.23 ± 0.10 ^a^	−13.3 ± 0.5 ^a^
*K. pneumoniae^Col-R^*	2370 ± 40 ^a^	0.27 ± 0.13 ^a^	−13.1 ± 0.6 ^a^

The data were expressed as mean ± standard deviation (SD) of 3–6 measurements; Means with different lowercase letters in the same column are significantly different at *p* < 0.05.

## Data Availability

The data presented in this study are available on request from the corresponding author.

## Supplementary Materials

The following are available online at https://www.mdpi.com/article/10.3390/ijms22137104/s1.
