# Supplementary material for: Evaluation of Chemical Changes in Laboratory-Induced Colistin-Resistant Klebsiella pneumoniae
Source: Int J Mol Sci. 2021 Jul 1;22(13):7104. doi: 10.3390/ijms22137104 (PMC8268070; doi:10.3390/ijms22137104)
Supplement: Supplementary file 1 [file ijms-22-07104-s001.zip › ijms-1274404-supplementary.pdf]

International Journal of
Molecular Sciences

MDPI

Supplementary Table S1

Figure 2	Starting wavenumber [cm^{-1}]	Ending wavenumber [cm^{-1}]	*Klebsiella pneumoniae*$^{Col-R}$ peak area [A · cm^{-1}]	*Klebsiella pneumoniae*$^{Col-S}$ peak area [A · cm^{-1}]
	3663	3115	192.6026	151.8776
	3102	3002	46.5249	42.8085
	2945	2884	32.2048	31.0923
	1719	1579	93.2991	82.4637
	1579	1474	71.3844	63.6642
	1459	1420	17.1443	16.4015
	1269	1181	11.9993	9.7758
	1181	908	101.1301	91.3327
	908	842	0.3504	4.383
**Figure 3**	**Starting wavenumber [cm^{-1}]**	**Ending wavenumber [cm^{-1}]**	**LPS$^{Klebsiella\ pneumoniae_Col-R}$ peak area [A · cm^{-1}]**	**LPS$^{Klebsiella\ pneumoniae_Col-S}$ peak area [A · cm^{-1}]**
	3684	2962	141.5303	183.245
	2962	2888	16.9926	21.2674
	2888	2831	11.2973	14.0268
	1800	1200	56.3114	77.5749
	1200	905	181.5496	166.7721
	905	870	8.6144	7.3886
**Figure 4**	**Starting wavenumber [cm^{-1}]**	**Ending wavenumber [cm^{-1}]**	***Klebsiella pneumoniae*$^{Col-R}$ peak area [I · cm^{-1}]**	***Klebsiella pneumoniae*$^{Col-S}$ peak's area [I · cm^{-1}]**
	3000	2820	3.3062	3.0529
	2579	2200	5.8778	5.2116
	2200	1774	13.5404	13.2205
	1774	1505	12.1232	11.4199
	1505	1339	7.4726	7.178
	1339	903	21.5799	19.3367
	903	650	12.2211	9.943
**Figure 5**	**Starting wavenumber [cm^{-1}]**	**Ending wavenumber [cm^{-1}]**	**LPS$^{Klebsiella\ pneumoniae_Col-R}$ peak area [I · cm^{-1}]**	**LPS$^{Klebsiella\ pneumoniae_Col-S}$ peak's area [I · cm^{-1}]**
	3028	2918	34.7454	42.5528
	2918	2789	31.3738	37.0259
	1674	1519	9.418	12.4515
	1519	1358	15.657	21.6373
	1164	969	19.0405	22.3885
	800	704	8.8463	10.1588
	458	399	17.6113	4.4542
	399	309	1.2378	8.7034
